# A workplace intervention to reduce alcohol and drug consumption: a nonrandomized single-group study

**DOI:** 10.1186/s12889-018-6133-y

**Published:** 2018-11-20

**Authors:** Montse Gómez-Recasens, Silvana Alfaro-Barrio, Lucia Tarro, Elisabet Llauradó, Rosa Solà

**Affiliations:** 1 Universitat Rovira i Virgili, Facultat de Medicina i Ciències de la Salut, Functional Nutrition, Oxidation, and Cardiovascular Diseases Group (NFOC-Salut), Health Education and Promotion, Reus, Spain; 2 Medical service of Fomento de Construcciones y Contratas, Delegación Catalunya II, 43007 Tarragona, Spain; 3 Eurecat, Centre Tecnològic de Catalunya, Unitat de Nutrició i Salut, Reus, Spain; 4 Hospital Universitari Sant Joan de Reus, Reus, Spain

**Keywords:** Health, Healthy living, Labor sphere, Risky behaviors, Habits, Drug prevention, Drugs, Work, Health behavior surveillance

## Abstract

**Background:**

The consumption of alcohol and other drugs causes social and health problems in industrialized societies. Furthermore, alcohol and drug consumption in the workplace is associated with work accidents, absenteeism and low productivity. The aim of the current study is to reduce alcohol and drug consumption among workers in the service industry and, as a secondary aim, to improve their healthy habits through the reduction of alcohol and other drug consumption in their leisure time.

**Methods:**

This nonrandomized, single-group study was conducted in 12 work centers. The intervention began in 2009 and emphasized 1) health promotion and health monitoring, which included a) alcohol and drug awareness and b) the evaluation and monitoring of alcohol and drug consumption through a semistructured interview designed to assess risky consumption; urine tests aimed at detecting alcohol, cannabis and cocaine use; an Alcotest based on expired air to test for the recent consumption of alcohol and a saliva exam to test for the recent consumption of six drugs; and 2) secondary prevention if risky consumption was identified. Risky alcohol consumption was defined as the ingestion of more than 28 standard drink units (SDUs)/week among men and more than 17 SDUs/week among women (taking into account both work and leisure time). Drug consumption was considered risky consumption.

**Results:**

A total of 1103 workers participated, and each received 5 h of awareness training. Those who presented with risky consumption received secondary prevention training. The prevalence of risky alcohol consumption decreased by 4.1% (baseline: 14.7% reduced to 10.6% in the first year; *p* = 0.001), a reduction that was maintained over a 3-year follow-up period.

**Conclusion:**

A comprehensive program of worker health surveillance that involves stakeholders and includes monitoring can be a means of potentially improving compliance with workplace promotion programs, resulting in the facilitation of such beneficial, desired behavior change in areas such as alcohol and drug consumption.

**Electronic supplementary material:**

The online version of this article (10.1186/s12889-018-6133-y) contains supplementary material, which is available to authorized users.

## Background

The consumption of alcohol and other drugs causes substantial social and health problems in industrialized societies [1]. Companies, like other social organizations, are not immune to the effects of alcohol and other drug consumption [2].

Alcohol consumption increases the risk of mortality and cancer, suggesting that policies to address alcohol consumption should be reviewed [3]. Europe has the highest alcohol consumption rate per habitant worldwide, and this value continues to increase [4, 5]. Specifically, Europeans consume approximately 12.5 l of alcohol per capita annually, whereas Spaniards consume 8 l per capita annually [6]. Alcohol consumption is one of the three major risk factors for premature death in Europe, in addition to tobacco use and overweight/obesity [7].

In 2014, 13% of Europeans reported cannabis use, and 2% reported cocaine use. Specifically, these consumption rates are highest in Spain, achieving levels of 17% and a 3.3%, respectively [8].

In Spain, alcohol was the substance consumed in the greatest quantities in 2015, according to diary reports, achieving a reported prevalence of 9.3% among 15- to 24-year-olds. Cannabis was the most prevalently consumed psychoactive illegal substance, showing a prevalence of 2.1% [9]. In 2015, the reported alcohol consumption rate in Catalonia was approximately 9.8%, with a risky consumption rate of 3.9%; the rates for cannabis and cocaine consumption were 2.5% and 0.1%, respectively [10].

The repercussions of substance consumption in the workplace include work accidents, absenteeism, work incapacities and decreased productivity [1]. In 2008, the Work International Organization recommended the implementation of political and work health programs to prevent alcohol and other drug consumption because it considers the workplace as an ideal venue for these programs to reduce alcohol and drug consumption and to promote health [11, 12]. Subsequently, the World Health Organization (WHO) developed the health environment criteria to encourage companies to adopt measures designed to prevent the consumption of alcohol or other drugs [13].

The European Workplace Alcohol (EWA) project, an integral program aimed toward higher risk members of the population, uses instruments to measure outcomes, provides instructions to facilitate treatment or referral to specialist services, and establishes efficacy systems to drive in situ treatment [14, 15]. Interventions that include the monitoring of variables such as risky alcohol consumption seem to be more effective in obtaining healthy behavior changes than isolated interventions that only account for sensitized and/or political improvements, for example, hanging posters with information or performing alcohol or drug tests during work hours [15].

The Catalonia Delegation II of the environmental area of Fomento de Construcciones y Contratas S.A. (hereafter, FCC S.A. Delegation) identified that employees demonstrated a higher rate of risky consumption of alcohol and other drugs than the reported risky alcohol consumption rate of 3.5% in the Catalan population.

## Methods

The present program aims to reduce the consumption of alcohol and other drug by service employees of the FCC S.A. Delegation in the workplace by implementing an integral program based on health promotion and a prevention intervention and, as a secondary aim, to improve healthy habits through the reduction of alcohol and other drug consumption during leisure time.

### Design

**Fig. 1:**
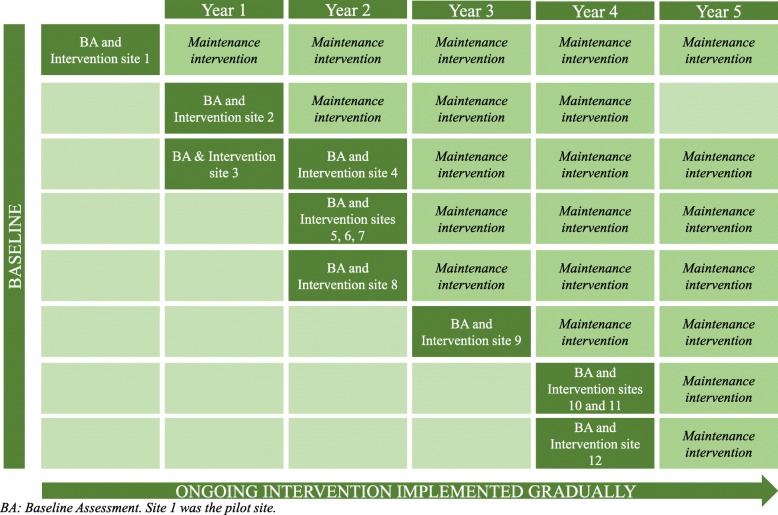
Gradual implementation of intervention (stepped wedge design)

This intervention, assessed in a nonrandomized single group, study aimed to promote health and prevent alcohol and drug consumption in the workplace and was conducted in 12 work centers. This workplace program was started in 2009, enrolling 240 employees in the pilot intervention in Reus. Afterward, the recruitment was done gradually until it included 12 FCC S.A. workplace delegations located across Tarragona and Lleida provinces of Spain so that the intervention could be implemented in all 12 FCC S.A. delegations by the company responsible. The 12 sites of FCC S.A. received the same intervention and the same monitoring by executive and occupational health service and medical service staff. The 12 sites were introduced gradually to the intervention; for logistical reasons, Reus was first, and Selsa was last (Fig. 1). The analysis considered the first incorporation of each employee in the present health promotion and prevention program as baseline data. The baseline data were generated at the following times: a) after the incorporation of a new worker, or b) at the first time of promotion and prevention program implementation. The workers first received a letter from the executive director of FCC S.A. with the presentation of the program. After the selection based on the inclusion criteria, the baseline assessment and program intervention were performed (Fig. 1).

This program obtained the approval of the security and health committees of all of the company worksites; members of the worksite unions agreed to participate and signed informed consent documents, which are now included in the collective bargaining agreement. The program design was approved by the Catalan public administration (Subdirección General de Drogodependencias de la Agència de Salut Pública de Catalunya del Departamento de Salud, Servicios Territoriales de Tarragona del Departamento de Trabajo). The confidentiality of the participants’ clinical information was strictly maintained according to the organic law 17/1999 ratified on December 13 regarding personal data protection. All reference data from the detection controls were collected from the medical service of the FCC S.A. Delegation.

### Inclusion criteria

To be eligible for inclusion, participants had to 1) have been an employee of the FCC S.A. Delegation with at least one year of service, 2) be ≥18 years old, and 3) have had at least one alcohol or other drug consumption registered through the work health surveillance program. Noncompliance with an inclusion criterion was considered grounds for exclusion.

### Intervention

After one year of previous work, the application of the prevention and action policies of zero tolerance and the intervention designed to prevent the consumption of alcohol and other drugs, the intervention program was developed and divided into 2 parts. Before its implementation, the intervention was explained as a professional training program that addressed the following:

- Executive and occupational health service training in promoting health [14].
- Medical service staff training in detection, brief intervention and motivational interviews to ensure the reliability among medical staff (the primary medical staff receive the same training from the general vice president for Drug Addiction of the Agència de Salut Pública de Catalunya).

**Fig. 2:**
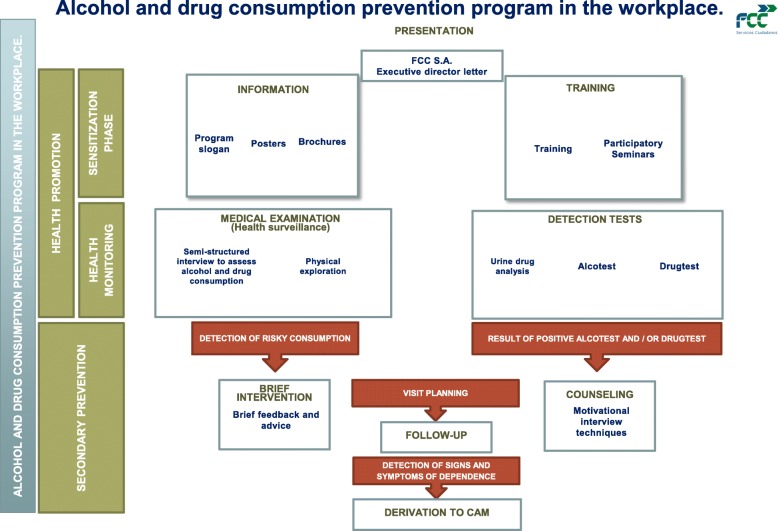
Alcohol and drug consumption prevention program in workplace

This program was initially designed to be comprehensive (Fig. 2).

#### A) First process: Health promotion and health monitoring

*A.1 Awareness phase:* Employees received different actions during the first year of enrollment in the program. These actions included the Occupational Health and Prevention Services of the FCC S.A. Delegation, the general vice president for drug addiction of the Agència de Salut Pública de Catalunya, and authority and security agents.

*A.1.a) Information:*

Each employer received a personal letter from the Executive of the FCC S.A. Delegation regarding program implementation.

Posters with images and messages of the program were designed and showed alcohol and/or drugs risks during work hours and extra-work hours. This message was positive and not coercive regarding consumption (Additional file 1).

*A.1.b) Training:*

All employees received 5 h of training in methods designed to change behaviors and reduce alcohol and drug consumption through the active encouragement of participants in discussions of real cases in groups of 25 employers who expressed their doubts and prejudices.

Training topics

○ Surveys of employer opinions regarding alcohol and/or other drug use, as well as the relevance of program implementation.

○ Conflicts related to the consumption of alcohol and/or other drugs.

○ Explanations of the alcohol and drug prevention programs in Catalonia and the conception of the present program.

○ Relevance of the role of each employer in the program.

○ Behavior during conflicts and the procedures associated with consumption during work hours at both relationship and work levels.

○ Program internal regulations: The consequences of tests meant to detect the use of alcohol and/or other drugs at work.

*A.1.c) Participation in workshops outside of work:*

Different activities/workshops were provided during work hours as expositions and technical workshops concerning the present program, but some activities were conducted outside of the workplace during work hours.

*A.2. Evaluation and health surveillance:*

At baseline and in the consecutive years after conducting the initial intervention, the Occupational Health Service of the FCC S.A. Delegation assessed the results to identify employees exhibiting risky consumption of alcohol and/or other drugs (during work and leisure time).

*A.2.a) Medical examination:*

A semistructured interview was conducted by a physician and a nurse with the purpose of evaluating and monitoring the alcohol and drug consumption of each employee (assessed in standard drink units (SDUs), where 1 SDU = 10 g of alcohol) and/or other drugs during or outside of work, in the past week. Physician and nurse staff were previously trained and standardized regarding semistructured interviews to ensure reliability. In addition, signs related to alcohol and/or drug consumption were assessed. This semistructured interview had questions about how many SDUs of alcohol and drug were consumed inside or outside of work hours and on weekdays or weekends to determine the scope of alcohol abuse. The obtained information was treated confidentially by the medical service and was not remitted to responsible FCC S.A. The results of the interview did not influence the relationships of the employees with the company.

Furthermore, detection tests (Alcotests, drug tests and urine tests) were performed among employees under different circumstances (randomly, for suspected acute poisoning, for work accidents and for planned detection).

#### B) Second process: Secondary prevention

Secondary prevention was conducted by the Occupational Health Service of the FCC S.A. Delegation following their expert’s criteria personalized for each worker according to their risky consumption of alcohol and/or other drugs (during work and leisure time). The secondary interventions were as follows:

*B.1 Brief intervention:* Risky alcohol and/or drug consumption was detected in employees using a semistructured interview; a brief 10–15 min intervention based on objective negotiation strategies to change the behaviors associated with consumption patterns was implemented [16].

*B.2 Personalized advice:* Among employees with a positive drug test (i.e., Alcotest, drug test and/or urine test), a motivational interview was performed to explore and solve doubts that would enable the employee to agree to reduce his/her consumption.

*B.3 Personalized follow-up assessment:* Among employees who received the brief intervention and/or personalized advice, a personalized follow-up assessment was conducted through a planned visit (when necessary), where the risk of consumption was assessed, or detection tests were used to confirm the employee’s capability to work.

*B4. Referral to the Center for the Attention and Monitoring of Drug Addictions (CAM):* Employees presenting with the signs and symptoms of substance dependence according to the International Statistical Classification of Diseases and Problems Related to Health, 10th review (ICD 10) of the WHO were referred to the CAM for diagnosis and multidisciplinary treatment.

### Outcomes

The principal outcomes were a) risky alcohol consumption (more than 28 SDUs/week in men and 17 SDUs/week in women), as assessed by the semistructured interview of consumption habits [17] (taking into account global consumption, during work and leisure time), b) drug consumption (positive detection was considered as risky consumption), and c) total risky consumption (combination of risky alcohol consumption and drug consumption).

An Alcotest (Dräger Alcotest ® 6810 med, Madrid, Spain) greater than 0.0 units was considered a positive test across two measures of the alcohol content in exhaled air separated by a 10 min interval (Instrucción 07/S- 94 Dirección General de Tráfico, 2008). The drug test (Dräger drug test ® 5000, Madrid, Spain) was considered positive if any drugs (e.g., opiates, cocaine, tetrahydrocannabinol, benzodiazepines, amphetamines and methamphetamines) were detected in the saliva (Instrucción 07/S- 94 Dirección General de Tráfico, 2008).

Furthermore, the following outcomes were recorded and compared with the WHO standards: weight (kg) using the Roman scale, height (m) using the Lohman scale [18], body mass index (BMI) (kg/m^2^; categorized using the WHO thresholds) [19] and systolic and diastolic blood pressure (mmHg) [20].

### Statistical analyses

Continues variables are presented as the means ± standard deviations (SD), and categorical variables are presented as percentages. McNemar’s test was used to compare the categorical variables between the baseline measurements and after the intervention.

All data were analyzed using SPSS V.23.0 for Windows (SPSS Inc., Chicago, Illinois, USA). The level of statistical significance was set to *p* < 0.05.

## Results

**Fig. 3:**
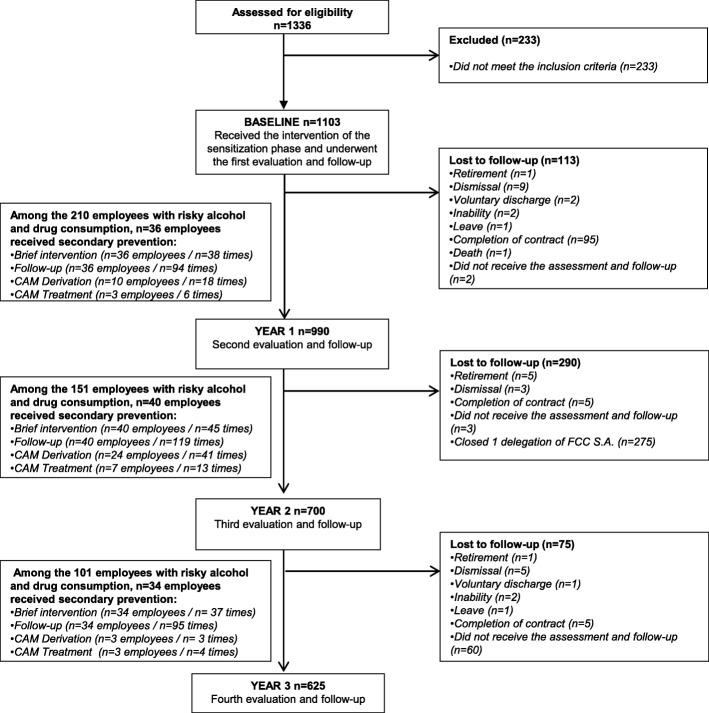
Flow diagram of the participants

One thousand one hundred and three employees participated in the program (Fig. 3), of whom 26% (*n* = 287) were female, and 74% (*n* = 816) were male. The mean age of the participants was 42.48 ± 10.44 years (Table 1).

**Table 1:** Employees’ baseline characteristics

Characteristic	Mean (±SD)	N (%)
Age	42.48 (±10.44)	
Weight (kg)	79.71 (±16.03)	
Height (m)	1.71 (±0.84)	
IMC (kg/m^2^)	27.30 (±4.82)	
Systolic blood pressure (mmHg)	131.6 (±18.01)	
Diastolic blood pressure (mmHg)	78.6 (±11.54)	
Women		287 (26)
Men		816 (74)
Categories of work		
Blue collar		1069 (96.9)
Elementary occupations		634 (57.5)
Cleaners and helpers		634 (57.5)
Plant and machine operators and assemblers		435 (39.4)
Stationary plant and machine operators		151 (13.7)
Drivers and mobile plant operators		252 (22.8)
Foreman of operators and drivers		32 (2.9)
White collar		34 (3.1)
Managers: administrative and commercial managers/production and specialized service managers		34 (3.1)
Risky consumption		
Alcohol		162 (14.7)
Drugs		75 (6.8)
Alcohol + Drugs		210 (19.0)

*SD* standard deviation, *N* participant number, *kg* kilograms, *m* meters, *mmHg* millimeters of mercury

Categories of work: Based on the International Labour Organization [21]

At baseline, 14.7% (*n* = 162) of employees presented risky alcohol consumption and 6.8% (*n* = 75) presented drug consumption. Overall, 19% (*n* = 210) of employees presented risky consumption of alcohol or drugs. The employees who presented risky consumption of alcohol and/or other drugs at baseline received the following specific secondary prevention measures, personalized depending on their characteristics: brief intervention (*n* = 36), follow-up (n = 36), referral to CAM (*n* = 10) and CAM treatment (*n* = 3) (Fig. 3).

Regarding risky alcohol consumption (during work and leisure time), a significant decrease of 4% was observed from baseline (14.7%; *n* = 162) to the first year (10.6%; *n* = 105; *p* < 0.001), and this change was maintained during the three-year follow-up assessment (*p* < 0.001). However, if the analysis of risky alcohol consumption was compared with the previous year, the tendency was to decrease in the second year, but from year 2 to year 3, the risky alcohol consumption was maintained at a stable level (*p* = 0.581) (Table 2). On the other hand, significant differences in risky drug consumption were not observed (Table 2), from year 1 to year 2 a significant reduction was presented (*p* = 0.039).

**Table 2:** Risky alcohol and drug consumption by participants in the program

Risky consumption	Baseline (*n* = 1103)	Year 1 (*n* = 990)	*P-value baseline vs. *year 1*	Year 2 (*n* = 700)	*P-value year 1 vs. *year 2*	Year 3 (*n* = 625)	*P-value year 2 vs. *year 3*	*P-value baseline vs. *year 2*	*P-value baseline vs. *year 3*
Alcohol % (*n*)	14.7 (162)	10.6 (105)	***< 0.001***	9.3 (65)	***0.002***	10.7 (63)	*0.581*	***< 0.001***	***< 0.001***
Drugs % (*n*)	6.8 (75)	6.6 (65)	*0.332*	6.7 (47)	***0.039***	6.9 (43)	*0.754*	*0.143*	*0.108*
Total % (*n*)	19 (210)	15.3 (151)	***< 0.001***	14.4 (101)	***0.001***	14.9 (93)	*0.424*	***< 0.001***	***< 0.001***

*p-value was calculated with McNemar’s test

Bold text indicates significant *p* values

An analysis of the results for risky alcohol consumption together with the consumption of alcohol and other drugs based on the work center revealed significant decreases in the Reus, Consell del Tarragonès, ECOBP and Tarragona centers (Table 3). Participants in the Reus center (a pilot center that included a 5-year follow-up period) presented a higher combined rate of risky alcohol and drug consumption at baseline, reaching 30.2% (*n* = 73), than that of other centers. Risky alcohol consumption decreased by 15% at the same center (from 26.4 to 11.4%, *p* < 0.001) and decreased to 6.1% over the 5 year follow-up period (*p* < 0.001) (Table 4).

**Table 3:** Risky alcohol and drug consumption by program participants stratified by work center

Work centers	Risky consumption	Baseline	Year 1	Year 2	Year 3
Reus (*n* = 242)	Alcohol % (*n*)	26.4 (64)	11.4 (25)*	9.3 (18)*	9.2 (17)*
	Drugs % (*n*)	9.5 (23)	9.5 (21)	8.8 (17)	9.7 (18)
	Alcohol + Drugs % (*n*)	30.2 (73)	19.1 (42)*	17.6 (34)*	14.6 (27)*
Consell (*n* = 63)	Alcohol % (*n*)	17.5(11)	5.7 (3)*	7 (3)	7.1 (3)
	Drugs % (*n*)	6.3 (4)	5.7 (3)	7 (3)	4.8 (2)
	Alcohol + Drugs % (*n*)	20.6 (13)	7.5 (4)*	9.3 (4)*	9.5 (4)*
Ecobp (*n* = 80)	Alcohol % (*n*)	16.3 (13)	15.7 (11)	9.7 (6)*	10.2 (6)*
	Drugs % (*n*)	10 (8)	10 (7)	6.5 (4)	0
	Alcohol + Drugs % (*n*)	23.8 (19)	21.4 (15)	12.9 (8)*	8.5 (5)*
Jaume Oro (*n* = 43)	Alcohol % (*n*)	11.6 (5)	12.2 (5)	8.1 (3)	12.9 (4)
	Drugs % (*n*)	2.3 (1)	2.4 (1)	2.7 (1)	3.2 (1)
	Alcohol + Drugs % (*n*)	14 (6)	14.6 (6)	10.8 (4)	12.9 (4)
Lleida (*n* = 56)	Alcohol % (*n*)	7.1 (4)	7.1 (4)	4.7 (2)	4.7 (2)
	Drugs % (*n*)	14.3 (8)	14.3 (8)	11.6 (5)	11.6 (5)
	Alcohol + Drugs % (*n*)	19.6 (11)	16.1 (9)	9.3 (4)	14 (6)
Tarragona (*n* = 348)	Alcohol % (*n*)	15.5 (54)	14.9 (45)	11.4 (31)*	14.2 (33)*
	Drugs % (n)	6.9 (24)	6 (18)	5.9 (16)	7.3 (17)
	Alcohol + Drugs % (*n*)	20.4 (71)	18.9 (57)	16.2 (44)*	19.3 (45)*
Ute Segria (*n* = 11)	Alcohol % (*n*)	0	0	0	0
	Drugs % (*n*)	9.1 (1)	10 (1)	14.3 (1)	0
	Alcohol + Drugs % (*n*)	9.1 (1)	10 (1)	14.3 (1)	0
Vendrell (*n* = 53)	Alcohol % (*n*)	11.3 (6)	13.2 (5)	7.4 (2)	7.7 (2)
	Drugs % (*n*)	1.9 (1)	2.6 (1)	0	0
	Alcohol + Drugs % (*n*)	13.2 (7)	15.8 (6)	7.4 (2)	7.7 (2)
White Collars "Oficinas" (*n* = 21)	Alcohol % (*n*)	4.8 (1)	5 (1)	0	
	Drugs % (*n*)	0	0	0	
	Alcohol + Drugs % (*n*)	4.8 (1)	5 (1)	0	
Deltebre (*n* = 41)	Alcohol % (*n*)	2.4 (1)	2.5 (1)		
	Drugs % (*n*)	2.4 (1)	5 (2)		
	Alcohol + drugs % (*n*)	4.9 (2)	2.5 (2)		
Cleaners (*n* = 102)	Alcohol % (*n*)	2.9 (3)	3.1 (3)		
	Drugs % (*n*)	2 (2)	2.1 (2)		
	Alcohol + Drugs % (*n*)	3.9 (4)	5.2 (5)		
Selsa (*n* = 43)	Alcohol % (*n*)	0	4.7 (2)		
	Drugs % (*n*)	4.7 (2)	4.7 (2)		
	Alcohol + Drugs % (*n*)	4.7 (2)	7 (3)		

*p < 0.05 compared with baseline values with McNemar's test

**Table 4:** Risky alcohol and drug consumption by participants in the program at the Reus work center

Work centers	Risky consumption	Baseline	Year 1	Year 2	Year 3	Year 4	Year 5
Reus (*n* = 242)	Alcohol % (*n*)	26.4 (64)	11.4 (25)*	9.3 (18)*	9.2 (17)*	7.7 (13)*	6.1 (9)*
	Drugs % (*n*)	9.5 (23)	9.5 (21)	8.8 (17)	9.7 (18)	7.1 (12)	5.4 (8)
	Alcohol + Drugs % (*n*)	30.2 (73)	19.1 (42)*	17.6 (34)*	14.6 (27)*	13.1 (22)*	9.5 (14)*

*p < 0.05 compared to baseline values with McNemar's test

## Discussion

The present comprehensive program designed to promote healthy behaviors and prevent the consumption of alcohol and/or other drugs was implemented in the workplace and effectively reduced risky alcohol consumption in employees in the FCC S.A. Delegation. This comprehensive program corroborates findings from other health promotion, behavior surveillance and secondary prevention programs in the workplace, contributing tools and guidelines to improve employees’ health [1]. Moreover, as the results demonstrate, the intervention could be reinforced at year 2, to continue with the reduction of alcohol and drugs consumption.

Health surveillance programs in various companies supply access to a contractual continuity and exposure of all employees to preventive messages or programs offered through the company [14, 22, 23]. In our experience, health surveillance is an occupational health service that facilitates health promotion.

The present comprehensive program has been applied at different institutions, which has generated a coresponsibility with companies in terms of social awareness regarding the necessity of reducing alcohol and/or other drug consumption. As a result, the reduction in the risky consumption of these substances was addressed in an intersectional manner using low-cost strategies after considering employers’ opinions regarding methods to attain greater participation in program strategies. This intersectional method consolidated a commitment based on the guidelines of the FCC S.A. Delegation. Thus, the intersectional method facilitated the long-term efficacy of the program in preventing the risky consumption of substances in the workplace [23, 24], thereby enabling its sustainability [7, 25].

Alcohol and/or other drug prevention programs based on health promotion in the workplace must include numerous strategies [23, 25], as described in the present program.

A systematic review identified 10 interventional studies designed to reduce alcohol consumption in the workplace; 5 studies were counseling interventions, 4 were based on feedback or brief interventions, and 1 was based on a peer-led methodology (among employees). The studies based on brief interventions were more effective than those using other methods [26]. The present comprehensive program comprised a brief intervention including a secondary prevention intervention, and it showed consistent results according to the intervention documents in 12 work centers, suggesting the effectiveness of our methodology in reducing harmful drinking behaviors. Furthermore, according to Sieck and Heirich, interventions based on promoting health and preventing (through close monitoring, as in our study) alcohol consumption show greater employee participation rates and a greater reduction of risky alcohol consumption than other programs solely based on punitive measures [27].

In the present comprehensive program, employees did not refuse alcohol or other drug tests because these tests had also been conducted during the awareness phase. Thus, these controls enabled the objective verification of the decrease in the consumption of alcohol and/or other drugs during work hours.

The literature regarding Spanish interventions only includes two published articles, one in Port de Barcelona [24] and the other in Red Nacional de los Ferrocarriles Españoles [28], based on substance detection during work hours; moreover, neither described the effectiveness of these programs. A Swedish transportation company program based on a randomized controlled intervention study revealed that the administration of drug-use detection tests reduces risky alcohol consumption and has effects similar to a brief intervention or training sessions [29]. Nevertheless, more evidence regarding the implementation of this type of intervention in Spanish populations is needed.

One of the principal limitations of this study is the lack of a control group. Due to company policies, all of its workers formed part of the intervention group because the company commission considered it nonethical that some workers would not otherwise receive the intervention. However, based on our knowledge, few randomized controlled trial interventions applied in workplaces were published because of the difficulty of justifying that some employees do not receive the intervention. Moreover, another limitation could be assumed by the Hawthorne effect, understood as a reaction of the target population of a study (intervention or control) that modifies their behavior due to the influence of participating in it [30]. In our case, although our population received exactly the same intervention and monitoring, they could be influenced by this effect because they are aware of all of the aspects of the intervention and the study process. Another limitation is the attrition rate of the study. To clarify this point, a comparison of the baseline characteristics of retained employees at follow-up with those of lost employees at follow-up showed that at baseline, the retained employees presented a higher prevalence of alcohol and drug risk consumption than lost employees (Additional file 2). Therefore, the positive effects of the study could be attributed to the intervention effect and not to attrition rates. However, the vast majority of losses at follow-up were due to the closure of a Delegation, considering that the economic crisis affecting Spain over these years also had an effect on the personnel restructuring in the company. From 2008 to 2014, unemployment increased in Spain from 8.60 to 26.94%, with the highest percentage in the European Union [30, 31]. Thus, the decreased worker retention in the present program was not due to a fear of missing work, as the data were anonymous for the Executive director of FCC S.A. Furthermore, few programs have evaluated results in the workplace [14, 32], and a vast number of them have small sample sizes; therefore, we were unable to compare our results with similar studies, which was another limitation. One more limitation is the substantial variability in the questionnaires used to evaluate the risky consumption of alcohol and/or other drugs among the programs. As a consequence, their results are difficult to compare with those of international studies [33]. Additionally, the current intervention has been gradually implemented in different work centers. The last centers that implemented the current methodology presented lower rates of risky consumption of alcohol and/or other drugs than the first centers. This finding may be due to the communication among the employees at different centers, which has been described in school-based interventions as contamination for communication among schools [34, 35].

Based on the results of the present study, we postulate that the implementation of comprehensive programs designed to reduce the consumption of alcohol and/or other drugs enables companies to increase their competitiveness, productivity and corporate social responsibility [14]. Despite the current situation, the workplace is and will remain a suitable environment to conduct programs designed to prevent health problems [13].

## Conclusion

A comprehensive program of worker health surveillance that involves stakeholders and includes monitoring can be a means of potentially improving compliance with workplace promotion programs, resulting in the facilitation of such beneficial, desired behavior change in areas such as alcohol and drug consumption.

## Additional files

Additional file 1: Posters with images and messages of the program over alcohol and/or drugs risks designed by FCC S.A. and showed during work hours and extra-work hours the overall of employees. (PPTX 604 kb)

Additional file 2: Comparison of the baseline characteristics of retained and non-retained employees at follow-up. This comparison showed the retained employees presented a higher prevalence of alcohol and drug risk consumption than lost employees at baseline. (DOCX 16 kb)

## Abbreviations

CAM: Center for Attention and Monitoring of Drug Addictions
EWA: European Workplace Alcohol
FCC S.A.: Fomento de Construcciones y Contratas S.A.
SD: Standard Deviation
WHO: World Health organization
