# Supplementary figures and images for: A workplace intervention to reduce alcohol and drug consumption: a nonrandomized single-group study
Source: BMC Public Health. 2018 Nov 20;18:1281. doi: 10.1186/s12889-018-6133-y (PMC6247683; doi:10.1186/s12889-018-6133-y)

## Slide 1
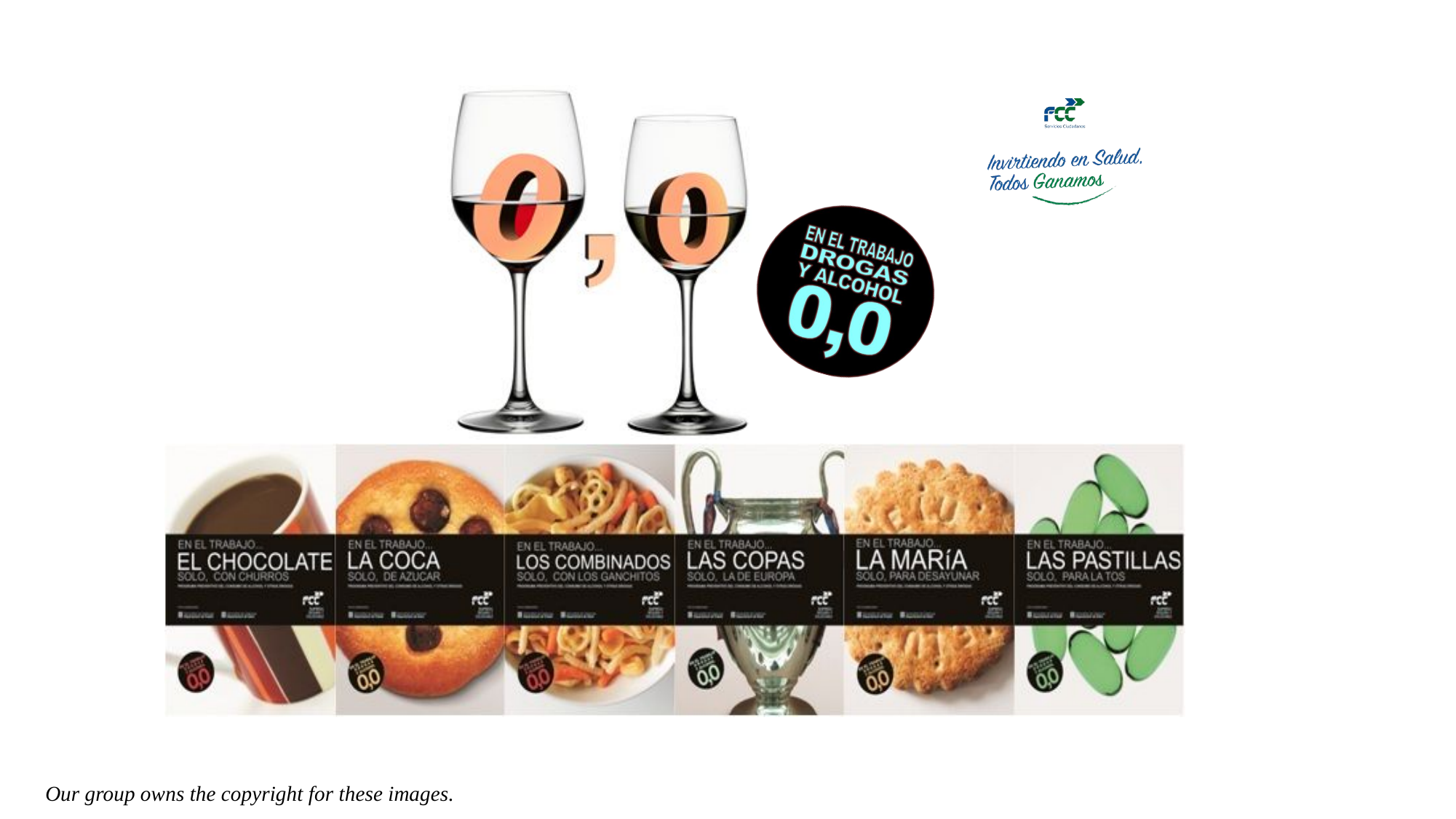

Our group owns the copyright for these images.

Supplement: Supplementary file 1 — Posters with images and messages of the program over alcohol and/or drugs risks designed by FCC S.A. and showed during work hours and extra-work hours the overall of employees. (PPTX 604 kb) [file 12889_2018_6133_MOESM1_ESM.pptx]
