# Supplementary material for: A workplace intervention to reduce alcohol and drug consumption: a nonrandomized single-group study
Source: BMC Public Health. 2018 Nov 20;18:1281. doi: 10.1186/s12889-018-6133-y (PMC6247683; doi:10.1186/s12889-018-6133-y)
Supplement: Supplementary file 2 — Comparison of the baseline characteristics of retained and non-retained employees at follow-up. This comparison showed the retained employees presented a higher prevalence of alcohol and drug risk consumption than lost employees at baseline. (DOCX 16 kb) [file 12889_2018_6133_MOESM2_ESM.docx]

**Additional file 2** Baseline alcohol and drug consumption among retained and lost participants

|  | Retained participants | Lost participants | p-value |
| --- | --- | --- | --- |
| Alcohol consumed % (n) | 19.8% (124) | 7.9% (38) | <0.001 |
| Drugs consumed % (n) | 8.3% (52) | 4.8% (23) | 0.022 |
| Alcohol + drug consumption % (n) | 25.1% (157) | 11.1% (53) | <0.001 |

*p<0.05
